# Assessing the Intention, Attitudes, and Social Influences on COVID-19 Preventive Behaviors Among Non-rural Black and Rural Appalachian White Populations: A Faith-Based Community Study

**DOI:** 10.13023/jah.0402.05

**Published:** 2022-07-01

**Authors:** Maria L. Gomez, Tofial Azam, Jean Edward, Hannah Bowman, Lovoria B. Williams

**Affiliations:** University of Kentucky, maria.gomez@uky.edu; University of Kentucky; University of Kentucky; University of Kentucky; University of Kentucky

**Keywords:** COVID-19, Public Health Preventive Behaviors, Appalachian, African American/Blacks, Theory Planned Behavior, Churches

## Abstract

**Introduction:**

The COVID-19 pandemic has had detrimental impacts in non-rural Black and rural Appalachian populations. Yet despite the pandemic’s magnitude, there is a scarcity of research exploring potential influences of attitudes and social influences within these populations on their adherence to COVID-19 public health preventive behaviors.

**Purpose:**

This study examines the intention, attitudes, and social influences to adhere to COVID-19 preventive behaviors among non-rural Black and rural Appalachian congregants in Kentucky by integrating the Theory of Planned Behavior (TPB).

**Methods:**

Secondary analysis of cross-sectional data was used to assess the association between the TPB constructs and four key public health behaviors: obeying a stay-at-home order, social distancing, good hygiene practices, and wearing a mask in public. Generalized estimating equation-type logistic regression models were fit for all binary outcomes.

**Results:**

A total of 942 respondents completed the survey. Eighty-nine per cent were older than 36 years, and 73% were female. Of the respondents who were White, 97.7% lived in rural Appalachia Kentucky, and of those who were Black, 93.5% lived in non-rural Kentucky. Attitude towards the behavior was negatively associated with the stay-at-home order ( p=0.003). Both attitude toward the behavior ( p<0.001) and the subjective norm ( p=0.025) were negatively associated with mask wearing. Perceived behavioral control was positively associated with mask wearing ( p=0.023) with non-rural respondents more likely to wear a mask than rural ones ( p<0.001). None of the TPB constructs showed significant association with hygiene practices or with social distancing.

**Implications:**

This study provides further insight into the cultural and societal influences that intersect during a global pandemic. The intention to comply with public health recommendations may vary at favorable and unfavorable levels. The results lend support to the importance of designing effective, culturally tailored communication for future public health preparedness.

## INTRODUCTION

The COVID-19 pandemic has had a disproportionate impact on Black/African American and rural Appalachian communities in the U.S.^1^,^2^ Roughly 180 out of every 100,000 Black Americans have died from COVID-19, a rate higher than for other racial/ethnic minority groups.^3^ Moreover, rural Americans are twice as likely to die from COVID-19 infection,^4^ at a 337.42 death rate per 100,000 population compared to 259.74 per 100,000 in urban areas.^5^ Along with pre-existing health conditions, place of residence has played a significant role in limiting access to care and COVID-19 resources for rural, Appalachian residents.^6^ Without access to adequate healthcare and public health resources to mitigate COVID-19, vulnerable populations such as Black and rural Appalachians will continue to be at an increased risk of COVID-19 infection, hospitalization, and death, as well as related complications, such as long COVID or post-COVID conditions.^7^–^9^

Despite the pandemic’s magnitude, research is scarce regarding the extent of social influences on adherence to public health recommendations (such as mask wearing) to prevent transmission of COVID-19.^10^,^11^ Ban Vabel et al. (2020) provide insight on the social influence context during pandemics, noting that the factors that influence people’s behavior include social norms, social inequalities in access to resources, culture, and attitudes toward government recommendations.^10^ Social norms have been described as “customary codes of behavior in a group or culture, together with the beliefs about what those codes mean.”^12^ Thus, these specific codes or norms can be considered as normal standards in our studied population.

Some studies conducted early in the pandemic have shown lower public knowledge of the health threat of COVID-19 infection and lower intention to comply with public health mitigation recommendations among certain adult populations, including those with chronic conditions.^13^–^15^ Notably, many of these studies had limited representation of racial- and ethnic-minority populations. Similarly, few studies exist on the impact of COVID-19 on Appalachian populations and their intent to comply with public health recommendations.^16^,^17^ In a nationally representative survey of 604 African Americans, 72% of respondents reported the intent to comply with hand washing, 67% with social distancing, and 65% with wearing a mask in public.^11^ In contrast, in a U.S. survey of more than 5,000 White respondents, 90% indicated the intent to comply with hand washing and 86% reported the intent to comply with social distancing/stay-at-home recommendations.^18^ Moreover, in a study of 5,009 American adults (60% White, 13.4% Black) that compared rural–urban population differences in COVID-19 preventive behaviors, researchers found that rural residents were 49% less likely to have worn a mask in public and to perform other preventive behaviors; rural residents accounted for 14.38% in this study, reflecting U.S. demographics.^16^ The considerably lower levels of adherence to public health recommendations place Black and rural populations at a higher risk of COVID-19 infection and even becoming severely ill due to well-documented persistent health disparities and social inequities in these populations.^1^,^2^ With the emergence of new COVID-19 variants, like Delta and Omicron, and the contentious social debate regarding the effectiveness of public health recommendations, examination of the intention, attitudes, and social influences on COVID-19 health behaviors among these underserved communities is timely.

Kentucky is one of 13 states in the Appalachian region, and it contains a large proportion of the rural communities there. Residents in Kentucky face persistent health disparities by race, ethnicity, geographic location, and socioeconomic status. About 40% of Kentucky’s population lives in rural areas, with 26% of them residing in Appalachia.^19^ This region is largely, though not exclusively, White—at 94.3% of the population.^19^ In contrast, minority populations are concentrated in the state’s metropolitan areas, such as Louisville, Lexington, and Bowling Green.^20^ Although Black/African American individuals only comprise about 8.5% of the total population in Kentucky, they represented 21% of all COVID-19 related deaths in the initial months of the pandemic.^21^ Initially, the virus spread rapidly in large urban areas of Kentucky, but by fall 2020, the number of cases in rural areas began to surge, posing challenges to timely healthcare treatment.^22^

The church plays a significant role in providing support during times of personal and psychological stress. Evidence indicates that church attendance and religious affiliation improve general well-being across multiple populations.^23^,^24^ The church has served as an integral partner in promoting health among its congregants. Church-based programs include promotion of HIV education and testing among racial and ethnic minorities,^25^ mental health promotion,^26^ and diabetes prevention among black congregants.^27^ Given the socially rich environments within churches, faith-based platforms present an opportunity to examine the social influence on COVID-19 prevention behaviors. For this reason, the study utilized partnerships developed with the leaders of churches^27^,^28^ in Appalachia and central Kentucky,^29^ to target recruitment of non-rural Black and rural Appalachian White populations.

The purpose of this study was to assess the intention, attitudes, and social influences related to COVID-19 preventive behaviors among two groups—nonrural Black and rural White Appalachian church congregants in Kentucky—by utilizing the Theory of Planned Behavior (TPB) framework.

### Theory of Planned Behavior (TPB) Framework

The TPB framework examines an individual’s intention to engage in a specific behavior.^30^ It posits that intention is influenced by three main constructs: (1) attitude toward the behavior; (2) subjective norms; and (3) perceived behavioral control. Attitude toward the behavior refers to the degree of positive or negative feelings toward performing a certain behavior. The subjective norms construct measures the extent of social influence to perform the behavior. In this context, subjective norms (or social pressure and influence from close networks) can play a fundamental role in an individual performing a behavior. The perceived behavioral control construct refers to how easy or difficult it is to perform the behavior of interest. The TPB framework has been used widely to investigate a person’s intention to perform specific behavior(s).^31^,^32^ The strength of this model is the prediction of intention, as an individual’s intention is motivated by internal as well as external factors.^33^ During the pandemic, external factors, such as influences from friends, family, and society, have been significant and could be shaping response to COVID-19-specific public health recommendations.

## METHODS

The study employed secondary data analysis of cross-sectional survey data collected from a convenience sample of 942 participants. The results of the parent study are published elsewhere.^34^ That study examined the impact of COVID-19 on the health, financial, and psychological consequences of Black non-rural and rural White Appalachian church congregants in Kentucky. Participants were invited by their pastors or church leaders to participate voluntarily in an anonymous survey. Due to social distancing restrictions, the survey was administered electronically with the Research Electronic Data Capture (REDCap) tool between May and September 2020, a critical period in the pandemic. There were two key eligibility criteria: age greater than or equal to 18 years and identification as a church member or church leader/pastor from (1) a predominantly Black church in non-rural central Kentucky, or (2) a rural church in Appalachian Kentucky. Congregants from fifteen churches in Appalachia and nine in central Kentucky participated. Based on the membership sizes of the churches and knowledge of technological gaps among the Appalachian region (i.e., the lack of wireless internet access), the target number of survey participants was 1,000. With this view, 942 respondents represented a high response rate, at 94%. The findings indicated delay in medical care and higher levels of anxiety and depression among the entire sample. Appalachian congregants were more likely to suffer financial constraints. Additionally, 33% of respondents indicated no intention to receive the COVID-19 vaccine. This study was approved by the University of Kentucky Office of Research Integrity in May 2020.

### Public Health Preventive Practices

Building on that previous work, this analysis examined the association of the TPB constructs in predicting the intent to perform four public health recommendations: (1) following stay-at-home orders; (2) social distancing; (3) practicing good hygiene; and (4) wearing a face covering or mask. Table 1 presents the questions related to preventive practices asked of participants. Because the frequency of responses in some of the groups was too small, they were pooled and grouped into a single bucket. For example, for the item asking about adherence to stay-at-home orders, a dichotomous response was created with responses “stay-at-home every day” and “not every day.” For the social distancing recommendation, a dichotomous response was created by combining responses in the “less than normal” and “not less than normal” categories. For the practicing hygiene outcome, a dichotomous response was created with responses “all of the time” and “not all of the time.” For the recommendation to wear a mask, a dichotomous response was created with frequencies of “all of the time” and “not all of the time.”

Eleven statements were established as the attitudes, social influences, and perceived behavioral control variables, as shown in Table 2. Five questions probed the attitude toward the behavior construct, three questions examined social norms or social influences, and three explored perceived behavioral control. All responses were rated on 4-point Likert scale of 1 (strongly disagree) to 4 (strongly agree). Summary scores for each construct were calculated as the average of the responses for the statements under the model.

### Data Analysis

Demographic characteristics were collected as categorical variables, frequencies, and percentages. A chi-square test was used to assess the association between demographic characteristics with respondents’ geographical locations. Cochran-Mantel-Haenszel chi-square based on modified ridit scores was used to assess the association between demographic variables with more than two levels and geographic location. Composite scores for the TPB constructs were created and treated as continuous outcome variables in the analysis. Generalized estimating equation (GEE)-type logistic regression models were fit for all the binary outcomes. For each model, TPB constructs and geographical location were included as covariates. A random effect of church was utilized in these models to account for the possibility of clustering due to the study design. Small sample-bias-corrected standard errors were utilized to ensure the validity of inference.^35^ Statistical analyses were performed using SAS version 9.4 (SAS Institute, 2015) and all tests were two-sided with statistical significance defined as *p*<0.05.

## RESULTS

### Socio-Demographic Characteristics

The demographic characteristics are presented in Table 3. A total of 942 congregants responded. The majority were female (73%) and greater than 36 years of age. Fifty percent of the respondents self-identified as Black or African American and 48% as White. The majority of the White congregants lived in rural Appalachian Kentucky (97.7%), and most Black congregants lived in nonrural central Kentucky (93.5%). Most of the respondents were married, with at least some college education, and earning more than $50,000 annually. Significant differences were noted in gender, race, education, and income between the two groups. Regarding working status, 35.4% of respondents were essential workers during the pandemic. About 60% of the sample was able to work from home, with non-rural Black congregants more likely to work from home (45% versus 34%, *p*=0.003).

Table 4 presents results from the multilevel logistic regression models examining the effect of the TPB constructs on congregants’ COVID-19 preventive behavior, adjusted for geographic locations. Results show that attitude toward the behavior construct is negatively associated with the stay-at-home order every day during the pandemic (OR=50, CI: 0.32–0.79, *p*=0.003) adjusted for all the other covariates in the model. This indicates that for one unit increase in attitude towards the behavior, the likelihood of congregants’ abiding by the stay-at-home order every day during the pandemic is decreased by 50%, adjusted for the other covariates in the model. Attitude toward the behavior (OR=0.29, CI: 0.17–0.48, *p*<0.001) and the subjective norms or normative belief (OR=0.66, CI: 0.46–0.95, *p*=0.025) are negatively associated with congregants wearing masks every day. The perceived behavioral control construct is positively associated with congregants wearing masks every day (OR=1.58, CI: 1.06–2.35, *p*=0.023). Furthermore, congregants in non-rural central Kentucky are three times more likely to wear a mask every day than congregants in the Appalachian region, adjusted for the TPB constructs (OR=3.07, CI: 2.35–5.85, *p*<0.001). None of the TPB constructs showed significant association with practicing hygiene recommendations or social distancing outside when adjusted for other covariates in the model.

## DISCUSSION

This study assessed the intention, attitudes, and social influences to adhere to COVID-19 preventive behaviors among two populations that have experienced higher COVID-19 morbidity and mortality. Findings indicate that the TPB constructs were significantly associated with the stay-at home order and face mask recommendations. It was surprising to find a negative association between the attitude toward the behavior construct and the stay-at-home order among respondents. This finding may indicate that—based on the extent of favorable or unfavorable attitude—there is a degree of doubt with compliance with the stay-at-home order every day and the benefits of this recommendation to reduce spread of COVID-19. Stay-at-home orders are the most extreme public health measure during pandemics.^36^ Data indicate that the impact of stay-at-home orders is isolating and has had a negative effect on mental health, triggering anxiety and depression in the U.S. and globally.^36^–^38^ The negative effects on mental health during the pandemic have been even more devastating among rural populations and women.^17^,^37^ Thus, an attitude toward the stay-at-home order can be attributed to various factors, including the fact that many churches were closed during the height of the pandemic, which prevented social association among our sample of churchgoers.

Ajzen (1991) notes that attitudes toward the behavior can be influenced by the level of confidence or knowledge an individual has about the health threat.^30^ Having access to reliable information sources alleviates fears and doubts, and it also clarifies concepts; therefore, positively influencing the expected behavioral change is associated with greater likelihood of engaging in the desired behavior. However, in light of the COVID-19 pandemic, an important finding among this body of research is the politicization of public health recommendations.^39^ The previous factors may have had an effect on the expected behavior change. This study’s findings of lower attitude toward COVID-19 stay-at-home orders/recommendations in the total sample is consistent with research by Alobuia et al. (2020) who found that racial/ethnic minority populations in the U.S. had lower attitude scores to overall preventive behaviors, yet higher adherence as socio-economic status increased, suggesting pervasive disparities due to social inequities.^40^ A research study on rural–urban adults (n=2,982) conducted in summer 2020 reported similar findings regarding lower adherence to avoid contact with people outside the household among the rural group.^41^ In that study, the majority of respondents were White; rural-dwellers accounted for 18.8%, and those in urban places, 81.2%.

The results of this study also show that the attitude toward the behavior and the subjective norms constructs are negatively associated with mask wearing. The face mask recommendation has triggered a national discourse and spurred divergent opinions. Despite observational and epidemiological evidence supporting the efficacy of mask wearing in controlling the spread of COVID-19, its use has been among the most contentious public health recommendations at the global level.^42^ Across the U.S., including within Kentucky, anti-mask rallies erupted.^43^,^44^ Likewise, subjective norms—referred to as the influence exerted by social groups or a person’s close networks—can manifest a degree of either greater refusal or greater acceptance in performing a behavior. The results suggest a degree of negative influence to wear a mask. A social norm can be more effective when trusted representatives from the specific community embrace it. Research indicates that Republican or Democrat political preferences may have influenced differences during the health crisis,^39^ with Republicans less likely to be engaged in preventive behaviors.^45^ In our sample, the attitude toward the behavior and subjective norms constructs are significant predictors of the intention to perform mask wearing. These findings are consistent with previous research demonstrating that Black Americans’ compliance with mask wearing was limited to 65%^11^ and that rural residents in the U.S were less likely to stay engaged than urban counterparts in various protective behaviors, including the use of masks.^16^

Our findings indicate that the perceived behavioral control construct is positively associated with, and significantly predictive of, mask wearing. The positive association suggests that there are no barriers to perform the desired outcome. In this context, factors of accessibility, affordability, and availability of masks facilitates the behavior.^12^ Scientific evidence indicates that mask wearing in fact reduces COVID-19 transmissibility, and it is most effective when compliance is high.^42^

In our analysis, about 72% of respondents indicated wearing a mask all the time. This finding is similar to a previous study reporting that adult Americans’ adherence to mask wearing ranges from 65% to 84%.^46^,^47^ In spite of this, our results showed significant differences in the use of masks between the two groups, with non-rural Black congregants more likely to wear a mask than rural White Appalachians. This finding is consistent with a study of a larger sample of rural and urban residents.^16^ These results, coupled with the lower vaccination rate among rural populations, partly explain the high hospitalization and mortality rates among rural Kentuckians in August 2021,^48^ a crucial time due to the emergence of the Delta variant that caused more severe illness among the unvaccinated. Although Black Kentuckians better adhered to mask wearing to some extent, the related hospitalizations were dramatic during the first year of the pandemic. This can be attributed to well-documented underlying health conditions—such as diabetes, stroke, and heart disease—which are more prevalent among Black/African American populations.^49^

This study contributes significantly to the limited research on factors affecting the intention to practice recommended protective health behaviors during the COVID-19 pandemic. Yet it is not without limitations. Inherent to secondary data analysis, findings were limited by the study design and data collected in the parent project. For example, having four groups comprised of both non-rural and rural Black and White populations would have strengthened the study’s ability to examine whether the differences were related to geographic region (i.e., rurality versus urbanicity) or to race. Although findings were similar to those of other studies, data used in the parent study were collected from a convenience sample of church congregants; external validity is therefore limited. Furthermore, the survey design was self-reported, and so the responses are subject to social desirability, which, given the rich social environment, may be higher among churchgoers than others. The effect is that respondents may overreport or underreport behaviors and attitudes. Lastly, the nature of cross-sectional study designs limits assessment or change in behavior over time. Although the data were collected during the critical initial months of the pandemic, at the time of data collection, new COVID-19 variants, such as the severe Delta and highly transmissible Omicron, had not yet emerged, nor was the vaccine developed. In the interim, respondents’ behaviors may have changed due to changing dynamics and the prolonged duration of the pandemic. A limitation of the TPB is that the framework does not consider the length of time between the intention and the performance of the action,^12^ and an individual may be subject to change a decision based on numerous factors. These facts— though limiting—can be useful in providing a basis for future follow-up evaluations and strategic communication strategies targeting these two populations.

Finally, it is also important to note that there is a gap in research on the impact of COVID-19 among rural Black congregants. Black pastors in southern rural Mississippi have highlighted the importance of church involvement in addressing the impact of the pandemic and in further researching its effects.^50^

## IMPLICATIONS

A strong body of rigorous science indicates the effectiveness of mask wearing in mitigating COVID-19 transmission and subsequent illness.^42^ Therefore, lower participation in COVID-19 public health protective behaviors will continue to contribute to increased morbidity and mortality among populations that already experience higher COVID-19 disparities. To reach Black and underserved Appalachian populations, we recommend the use of culturally tailored community-level strategies and the engagement of coalitions and credible sources that include faith-based leaders.

In conclusion, attitudes, social norms, and perceived behavioral control significantly predicted the intention, at favorable and unfavorable levels, to follow stay-at-home orders and wear masks, two key public health recommendations. The analysis, which was guided by the TPB, provides important insights into the importance of evaluating the intersection of cultural and societal influences on COVID-19 prevention behaviors. Future research using the TPB may need to measure timing between the intention and the actual compliance with an expected health behavior. Results can be used to inform the development of effective evidence-based communication strategies to mitigate the devastating effects of COVID-19 on these disparate populations.

**SUMMARY BOX**

**What is already known about this topic?**

The research of public intent to comply with COVID-19 public health recommendations mitigations is limited. This is a new area of investigation, as studies are still ongoing and developing.

**What is added by this report?**

We examined intention to perform a behavioral response to COVID-19 at the population level among non-rural Black and rural Appalachian populations, two priority groups that have experienced higher health disparities. The study additionally emphasizes the role of the Church in addressing the impact of the pandemic. This study provides insight into the importance of cultural and societal influences that intersect during a pandemic and can be used to inform the development of effective evidence-based communication strategies to mitigate COVID-19 and subsequent public health emergencies.

**What are the implications for future research?**

The lower participation in COVID-19 public health protective behavior among populations like the two studied here may contribute to increased morbidity and mortality rates from the virus, especially given the populations’ extant underlying health conditions (e.g., higher rates of obesity, diabetes, and cancer). Future research on behavioral pandemic response should consider culturally tailored community-level strategies and the inclusion of other racial minority groups and sub-groups to reduce health disparities.

## Figures and Tables

**Table 1:** COVID-19 Public Health Preventive Behaviors

Outcome	Responses
**Stay-at-Home Order** On an average week, during the COVID-19 stay-at-home recommendation, other than to do essential activities (grocery shop, pick up medications, work) I stayed home:	○ None of the days ○ A few days (1–2 days) ○ Most days (3–4) days ○ Every day
**Social Distancing** On an average week, during the COVID-19 social distancing recommendation, my social interaction with people outside of my home was:	○ A lot less than normal ○ Somewhat less than normal ○ About the same as normal ○ More than normal ○ A lot more than normal
**Practicing Hygiene** During the COVID-19 pandemic, how often did you do the recommended pandemic hygiene, like hand washing frequently, avoiding touching your face, wiping down surfaces in your home?	○ All of the time ○ Most of the time ○ Sometimes ○ Rarely
**Wearing a Face Mask** During the COVID-19 pandemic, how often did you wear a face covering/mask when you were in public places?	○ All of the time ○ Most of the time ○ Sometimes ○ Rarely

**Table 2:** Attitudes, Social Influences, and Perceived Behavioral Control

Attitudes Toward Behavior	
1.	If I get COVID-19, it was meant to be
2.	Wearing masks won’t make a difference in lowering the spread of COVID-19 to others
3.	Social distancing helps lower the spread of COVID-19
4.	Even if the COVID-19 infection rate begins to rise, the country should reopen
5.	I trust the information on the news about COVID-19
**Subjective Norms/Normative Beliefs**	
6.	Most of the people that I know think that COVID-19 is not as serious as the news makes it out to be
7.	Most of my friends think that we should avoid gathering in groups during the COVID-19 pandemic
8.	I feel social pressure to wear a mask
**Perceived Behavioral Control**	
9.	I am confident that I could get a face mask to wear
10.	I am confident that I could get a COVID-19 test if needed
11.	I am confident that I could afford healthcare treatment for COVID-19 if needed

NOTES:

Likert Scale Responses: (1) Strongly Disagree, (2) Disagree, (3) Agree, (4) Strongly Agree

**Table 3:** Demographic Characteristics of the Study Sample (n=942)

Demographic Variables	Non-rural Central KY n (%)	Rural Appalachia n (%)	Total n (%)	*P*-value
**Gender**				
Female	370 (75.82)	299 (69.86)	669 (73.03)	**0.043** *
Male	118 (24.18)	129 (30.14)	247 (26.97)	
**Age**				
Younger than 36	54 (11.00)	51 (11.92)	105 (11.43)	0.254
36–55	176 (35.85)	132 (30.84)	308 (33.51)	
56–65	130 (26.48)	112 (26.17)	242 (26.33)	
66 and older	131 (26.68)	133 (31.07)	264 (28.73)	
**Race**				
White	18 (3.68)	417 (97.66)	435 (47.49)	**<0.001** *
Black/African	457 (93.46)	2 (0.47)	459 (50.11)	
American				
Other	14 (2.86)	8 (1.87)	22 (2.40)	
**Education**				
≤Some high school	7 (1.44)	26 (6.09)	33 (3.61)	**<0.001** *
High school graduate	56 (11.50)	101 (23.65)	157 (17.18)	
Some college	184 (37.78)	134 (31.38)	318 (34.79)	
College graduate	116 (23.82)	90 (21.08)	206 (22.54)	
Graduate degree	124 (25.46)	76 (17.80)	200 (21.88)	
**Income (annually)**				
< $25,000	72 (15.69)	76 (18.45)	148 (16.99)	**0.026** *
$25,000–$50,000	129 (28.10)	140 (33.98)	269 (30.88)	
> $50,000	258 (56.21)	196 (47.57)	454 (52.12)	
**Marital Status**				
Single	107 (21.93)	37 (8.67)	144 (15.74)	0.905
Married	233 (47.75)	315 (73.77)	548 (59.89)	
Separated, Divorced or Widowed	148 (30.33)	75 (17.56)	223 (24.37)	
**Primary Occupation**				
Service related	50 (10.46)	26 (6.19)	76 (8.46)	**<0.001** *
Professional	187 (39.12)	124 (29.52)	311 (34.63)	
Healthcare Industry	83 (17.36)	56 (13.33)	139 (15.48)	
Other	158 (33.05)	214 (50.95)	372 (41.43)	
**Essential Worker During the COVID-19 Pandemic**				
Yes	146 (36.23)	118 (34.50)	264 (35.44)	0.624
No	257 (63.77)	224 (65.50)	481 (64.56)	
**Able to Work from Home During the COVID-19 Pandemic**				
Yes	198 (44.70)	127 (34.51)	486 (59.93)	**0.003** *
No	245 (55.30)	241 (65.49)	325 (40.07)	

NOTES:

* *p*<0.05.

**Table 4:** Associations Between Theory of Planned Behavior Constructs with Behavioral Change by Geographic Location

Outcome Variables Covariates	Odds Ratios	95% CI
**Stay-at-home order**		
Attitude toward the behavior	0.50	**(0.32, 0.79)** *
Subjective norms/normative beliefs	1.20	(0.91, 1.57)
Perceived behavioral control	1.13	(0.88, 1.46)
Geographic Central KY	1.05	(0.70, 1.57)
**Social distancing**		
Attitude toward the behavior	0.65	(0.27, 1.54)
Subjective norms/normative beliefs	1.17	(0.78, 1.76)
Perceived behavioral control	1.19	(0.75, 1.89)
Geographic Central KY	0.69	(0.45, 1.06)
**Wearing a Face Mask**		
Attitude toward the behavior	0.29	**(0.17, 0.48)** *
Subjective norms/normative beliefs	0.66	**(0.46, 0.95)** *
Perceived behavioral control	1.58	**(1.06, 2.35)** *
Geographic Central KY	3.07	**(2.35, 5.85)** *
**Practicing Hygiene Recommendation**		
Attitude toward the behavior	1.17	(0.75, 1.82)
Subjective norms/normative beliefs	0.99	(0.78, 1.25)
Perceived behavioral control	1.05	(0.75, 1.48)
Geographic Central KY	0.45	(0.93, 2.27)

NOTES:

* Variables with significant association at *p*<0.05.
